# Candidate List of yoUr Biomarker (CLUB): A Web-based Platform to Aid Cancer Biomarker Research

**DOI:** 10.4137/bmi.s467

**Published:** 2008-02-09

**Authors:** Bernett T.K. Lee, Lailing Liew, Jiahao Lim, Jonathan K.L. Tan, Tze Chuen Lee, Pardha S. Veladandi, Yun Ping Lim, Hao Han, Gunaretnam Rajagopal, N. Leigh Anderson

**Affiliations:** 1 Bioinformatics Institute, 30 Biopolis Street, #07-01, Singapore 138671; 2 The Plasma Proteome Insitute, P.O. Box: 53450, Washington DC, 20009-3450, U.S.A

## Abstract

CLUB (“Candidate List of yoUr Biomarkers”) is a freely available, web-based resource designed to support Cancer biomarker research. It is targeted to provide a comprehensive list of candidate biomarkers for various cancers that have been reported by the research community. CLUB provides tools for comparison of marker candidates from different experimental platforms, with the ability to filter, search, query and explore, molecular interaction networks associated with cancer biomarkers from the published literature and from data uploaded by the community. This complex and ambitious project is implemented in phases. As a first step, we have compiled from the literature an initial set of differentially expressed human candidate cancer biomarkers. Each candidate is annotated with information from publicly available databases such as Gene Ontology, Swiss-Prot database, National Center for Biotechnology Information’s reference sequences, Biomolecular Interaction Network Database and IntAct interaction. The user has the option to maintain private lists of biomarker candidates or share and export these for use by the community. Furthermore, users may customize and combine commonly used sets of selection procedures and apply them as a stored workflow using selected candidate lists. To enable an assessment by the user before taking a candidate biomarker to the experimental validation stage, the platform contains the functionality to identify pathways associated with cancer risk, staging, prognosis, outcome in cancer and other clinically associated phenotypes. The system is available at *http://club.bii.a-star.edu.sg*.

## Introduction

Biomarkers are molecular signatures that can serve as early warning signs of disease, measure disease progression or monitor the physiological effects of therapeutic interventions in the treatment of disease. Biomarker discovery is often carried out by comparing normal and disease states by differential gene/protein expression and changes in metabolic profiles. The hope of finding new biomarkers for assessing cancer risk, detecting cancer at an early stage, subtyping tumors, selecting optimal therapies, and monitoring therapeutic response is the motivation behind substantial current investments in biomarker research. Success in this effort can yield revolutionary improvements in the management of cancer. However, despite considerable public and private resources invested in biomarker discovery over the past twenty years, and a plethora of candidate biomarkers (Pritzker, 2002), the results in terms of clinical impact have been disappointing. To date, very few biomarkers have been approved by the Food and Drug Administration (FDA) for use in cancer diagnosis and therapy (Anderson and Anderson, 2002). While technical limitations in detecting biomarkers (Diamandis, 2004) represents one reason for this shortfall, poor study design, population heterogeneity and methodological weaknesses in data collection and analysis (Baggerly et al. 2005; Ransohoff, 2005; Zhang and Chan, 2005) have led to the reporting of numerous candidate biomarker sets for a given disease that show little or no overlap between studies. This lack of replication has undermined confidence that truly disease-associated clinically useful biomarkers can be found.

While much progress is being made around the world, verification and validation of putative biomarkers is difficult even if the shortcomings listed above can be overcome. In particular, given limited capacity to undertake candidate verification studies, any effective means that prioritize candidate biomarkers based on biological insight would be very useful (Aebersold et al. 2005). Given that there is already a depth of published knowledge from various studies (not all of which are of the same quality) it would be good if there existed a central repository bringing together the wealth of information from the literature for the discovery of truly clinically useful biomarkers. It would also serve as a community-wide platform where biomarker groups could share and compare protocols and data. Such a database should ideally be platform neutral so that researchers working on proteomics, microarray or other expression-based experiments can use the system to upload, filter, share, compare and analyze the candidate biomarkers.

To this end, given that effective therapies are targeting specific cancer pathways, we have developed and deployed CLUB (Candidate List of yoUr Biomarkers), which enables users to discover and document such pathways related to a particular disease subtype (Aebersold et al. 2005). While our focus is on cancer, its basic framework can be easily modified for other diseases. Part of the data within CLUB is derived from peer reviewed journals and is updated on a regular basis by an experienced team of curators. The 1261 candidate cancer biomarkers compiled by Polanski and Anderson (Polanski and Anderson, 2006), for example and other data can now be found in the system and is available for users to conduct meaningful comparisons, gene enrichments, stratify and analyze biomarker interaction networks to come up with interesting hypothesis. The molecular interaction features within CLUB also provide users with a means to understand the biological basis of why certain biomarkers work. Along with information from the literature and the pathway interaction tool, potentially important features of candidate biomarkers related to drug targets, treatment outcome and response to therapy may be discovered.

The utility of CLUB differs from earlier biomarker databases. The Biomarker Database (BMDB) by Feng and coworkers (Feng et al. 2005) caters only to microarray analysis, has limited curated information from the literature, and does not obtain manually curated interaction data for pathway related analysis. The BioMarker Database (BioMarker DB) developed by Jubilant Biosystem Ltd. is a repository for curated biomarkers. The Biomarker Database funded by The Korea National Institute of Health (http://biomarker.cdc.go.kr:8080/About/AboutBD_en.jsp?m=BD) focused on depositing infectious disease related biomarker and UniPep (Zhang et al. 2006) is a resource for N-linked glycosites from human serum biomarker discovery. While these tools are useful resources, CLUB is targeted instead towards community-wide participation in the aggregation, comparison, annotation and use of multiple biomarker candidate lists. With the capability to make comparisons between gene lists and discover biologically meaningful interactions, biomarkers that enable early diagnosis, monitor therapeutic responses, estimate disease prognosis and predict chemotherapeutic responses and resistance could be discovered using the system. As CLUB focuses on the ability to make comparisons between gene lists, complex statistical analysis tools have not been implemented and users are advised to download the data for use in a separate statistical package. As CLUB continues to evolve with more user feedback, we envision that its user-friendly community—wide access approach will enable systemic collection of data sets from the community and facilitate the selection of candidate biomarkers for validation. By this evolving process it will help to close the gap between basic research and FDA-approved clinically useful biomarkers.

## Results

**Figure 1:**
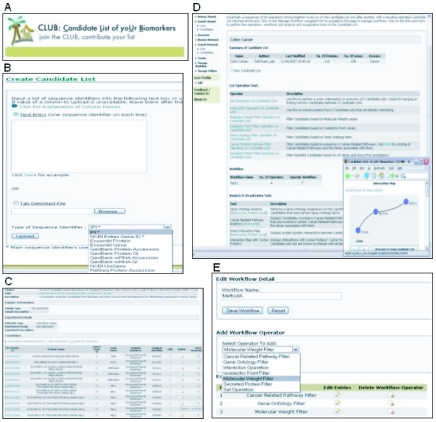
Sample screenshots from CLUB where user can conduct comparisons and various analyses on a list of candidate bio-markers (**A**) Logo of CLUB. (**B**) The Create List of Candidate Biomarker page. User may input biomarker based on a template or upload them directly from file with sixteen different recognizable identifiers. (**C**) The View Candidate List page where information about individual candidate biomarkers can be found. (**D**) Candidate Manipulations page where various analyses can be conducted. (**E**) Workflow page where user may customize and combine commonly used sets of operations to run them in a series.

**Figure 2:**
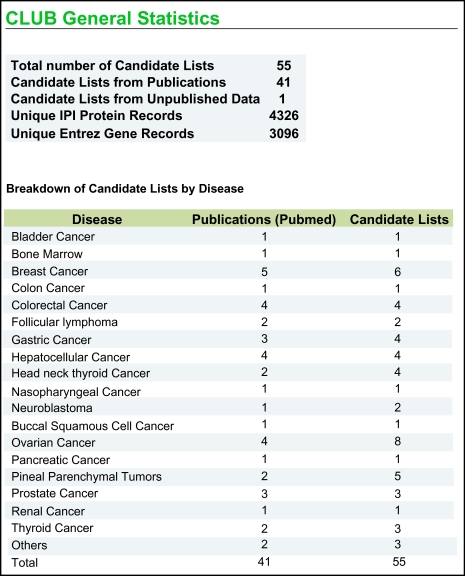
General Statistics of the number of candidate gene and protein biomarkers in CLUB Details of 55 candidate lists of various cancer-types containing 4326 distinct International Protein Index protein records and 3096 distinct Entrez Gene records have been curated in CLUB.

CLUB was designed to be platform neutral and thus is available to all as a web service. Users can access the service via a standard web browser, with some of the visualization components requiring the use of a Java plugin, which is available for most platforms (Fig. 1). A simple one-time registration is required to gain secure access to privately saved data. In order to initiate the community contribution process, we have embarked on a curation exercise to populate CLUB with an initial cancer-related dataset from publications using high-throughput technologies, especially those employing microarrays and mass spectrometry. As of June 2007, details of 55 lists of various cancer-types from 41 papers containing 4326 distinct International Protein Index (IPI) protein records and 3096 distinct Entrez Gene records have been curated and are available in CLUB’s public data section (Fig. 2). We are also working with International Cancer Biomarker Consortium (ICBC) to facilitate use of CLUB by the cancer biomarker community.

In CLUB, every biomarker candidate is assigned a corresponding NCBI Entrez Gene ID and/or IPI identifier, regardless of the accession number system in which identifications are originally reported. This consistent identifier forms the basis for comparing stored processed candidate biomarkers across all the different technology platforms, and overcomes a major barrier in comparing published biomarkers sets identified in different accession systems. Metadata of the candidate biomarker such as fold change, type of deregulation, and information about the candidate list such as sample type, experimental setups, category of candidate biomarker, stage of cancer, available publications etc. may also be entered into the system. These meta-data also serves as a means to compare, search and filter biomarker candidates.

Users may upload biomarker candidates and save them as a list of candidates with sixteen commonly used identifiers (Fig. 1B). The original uploaded identifiers will be mapped to a corresponding Entrez Gene ID and an IPI identifier. As heterogeneous data from various experimental platforms are compared in the system, details from the unprocessed raw data were omitted from the file upload section. Users may also include detailed unprocessed data and/or PubMed references via hyperlinks (Fig. 1C). Each individual candidate is also annotated with information from Swiss-Prot (Bairoch and Apweiler, 1997), Gene Ontology (Ashburner et al. 2000) and GenMAPP (Dahlquist et al. 2002). Lists of biomarker candidates (usually tied to a specific experiment or a group of candidates) and individual biomarker candidates may be searched, browsed, shared with fellow CLUB users, saved, exported (in Microsoft Excel format) and managed in folders. Users may also have an overview of the detailed information of their selected list of biomarker candidates. Information such as the type of disease, stage of the cancer samples, purpose and design of the experiments and other details about the biomarker candidates may be displayed at the overview page.

Recent therapeutic strategies are evolving to target cancer specific signal transduction and metabolic pathways and it is becoming important to document these pathways as powerful tools in cancer management (Aebersold et al. 2005; Gulmann et al. 2006). Hence, pathway analysis and pathway discovery of cancer subtypes are becoming increasingly important for the understanding of the pathophysiology of neoplasia and anticancer drug discovery (Cho, 2007; Efroni et al. 2007). CLUB facilitates analysis of this nature by incorporating manually-curated interaction data from BIND (Bader et al. 2003) and IntAct interaction database (Kerrien et al. 2007). Users may use an “interaction operator” to find how a list of candidate biomarkers interacts with another list (say apoptosis related proteins, oncogenes or tumor suppressors). Links to the references that describes the interactions found in the network generated are available. The interaction visualization tool may be used to see interacting or regulating genes or proteins within a given list.

A range of functionalities can be used to perform analysis on a candidate list or between candidate lists (Fig. 1D). Biomarker candidates may be filtered by protein molecular weight, isoelectric point, gene ontology, presence in cancer related pathways, or according to whether they are classified or annotated by Gene Ontology and Swiss-Prot as a secreted protein. User may also use boolean set operations to compare candidate biomarkers from any 2 candidate lists. For example, it might be reassuring when users find that their fresh observations appear repeatedly in similar independent studies. Users may also like to conduct a gene ontology breakdown on the biomarkers or find out which cancer related pathways the biomarkers in the candidate list belong to. Bar charts and histograms can also be used to display the distribution of molecular weight and isoelectric point values within the selected candidate list. These functionalities may act as individual modules where users may customize and combine commonly used sets of operations to run them in a series like a workflow on their selected set of candidate list (Fig. 1E).

## Discussion

**Figure 3:**
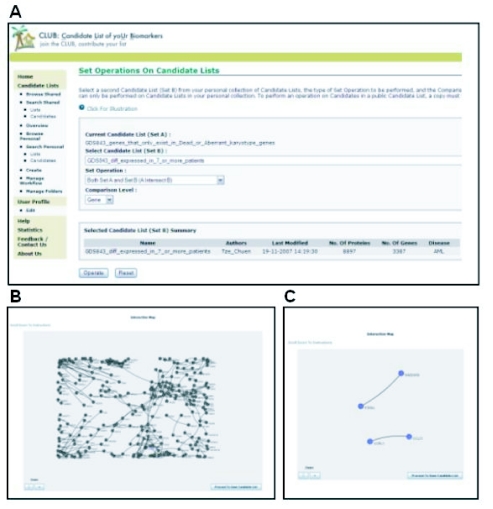
Screenshots from CLUB illustrating data filtering and analysis procedures to select potential prognostic markers from Bullinger et al.’s data (**A**) Filtering for highly represented diffentially expressed genes from the group of patients with poor prognosis. (**B**) Filtering for genes that correlate to one another via gene-gene interaction data. This procedure may provide biological insights to the gene list. (**C**) Interaction networks found only in the group of patients associated with poor prognosis.

To illustrate some of the functionalities of CLUB, we use it to analyze a public dataset from Bullinger et al. (Bullinger et al. 2004). The original data covers samples from 116 adults with Acute Myeloid Leukemia (AML, including 54 tissue samples derived from the bone marrow) which were analyzed by three separate platforms and classified in terms of their clinical outcome (dead or alive) and genotype variation (normal or aberrant karyotype). We took the 20 bone marrow derived samples analyzed using the GEO accession GDS843 microarray platform, leaving out the sample with an intermediate phenotype. The expression level for each gene is compared with the levels of Corticotropin Releasing Hormone (CRH) expression. Only genes with greater than 2-fold expression difference were included. We sought to identify potential prognostic biomarkers by choosing interaction network clusters that were only found in samples from dead patients and samples from living patients with aberrant karyotype. Differentially expressed genes that were highly represented in patients were selected to be analyzed. Henceforth, 3 gene lists were uploaded to CLUB: (i) the set of genes found in patients that were alive and have normal karyotype, (ii) the set of genes found in patients that were dead or have aberrant karyotype and (iii) the set of genes that were found differentially expressed in 7 or more patients. Using CLUB’s set operation (intersection) function, we filter for genes that were highly represented in the 2 groups of patients (Fig. 3A). Subsequently, with CLUB’s interacting function, gene-interaction networks were generated for the remaining genes that passed through the first criteria (Fig. 3B). In order to look for interacting networks that are specific to the group of patients with a poorer prognosis, we used CLUB’s set operation (“Set A but Not Set B”) to include all the saved genes found in the group that were dead or has an aberrant karyotype and excluded the genes that can be found in the group that were alive and have normal karyotype. Four potential prognostic biomarkers representing 2 interaction networks showed up from this series of procedures. The CCL21 (Small Inducible cytokine A21)—CCRL1 (Chemokine Receptor Type 11) interaction are known to play a role in neoplastic metastasis while the GADD45G (Growth Arrest and DNA-Damage-Inducible 45 Gamma) in the GADD45G-PTPRK (Protein Tyrosine Phosphatase Receptor type K) interaction is a known pro-apoptotic factor (Fig. 3C). These interaction network clusters represent candidate networks that are potentially specific to leukemia patients with well-advanced stage of cancer and therefore, a poorer prognosis for patients with these interaction networks. To find out if these candidates were listed as potential biomarkers in Polanski and Anderson’s (Polanski and Anderson, 2006) list of 1261 candidate biomarkers, we did an intersecting set operation with this list from CLUB. Two (GADD45G, CCL21) out of 4 candidates found in the 2 interaction network clusters can also be found in Polanski and Anderson’s list. These results illustrate the usefulness of using analysis tools from CLUB to shortlist biomarkers that merit further clinical evaluations in AML patient prognosis. The network clusters may also act to correlate one candidate biomarker to another and may therefore assist researchers in the gene-enrichment and gene-filtering process.

As research is being conducted to understand why certain biomarkers work in the clinic, we hope to identify characteristics of clinically useful biomarkers and eventually come up with a scoring system that ranks their likelihood of becoming a successful candidate. In future, CLUB may also include genetic, glycomic, lipidomic, metabolomic and other expression based biomarkers for analysis. We also intend to design CLUB to make it more useful and relevant for clinicians. Researchers and clinicians with interests in the field are invited to submit relevant candidate cancer biomarkers and/or suggest improvements to the database. CLUB is available at http://club.bii.a-star.edu.sg/.
